# Using proximity labelling to investigate astrocytic protein profile changes in response to amyloid pathology in a mammalian model of Alzheimer’s disease

**DOI:** 10.1002/alz.090638

**Published:** 2025-01-03

**Authors:** Perlina Desai, Lorena Arancibia Carcamo, Bart De Strooper

**Affiliations:** ^1^ UK Dementia Research Institute @ University College London, London United Kingdom; ^2^ The Francis Crick Institute, London United Kingdom; ^3^ VIB ‐ KU Leuven Center for Brain & Disease Research, Leuven Belgium; ^4^ KU Leuven, Leuven Brain Institute, Leuven Belgium

## Abstract

**Background:**

Astrocytes are a numerous and diverse glial subtype specialised to carry out distinct roles involving maintaining homeostasis and effective functioning of the nervous system. To do so effectively, they respond to and secrete various proteins. In addition, astrocytes have been linked to Alzheimer’s disease (AD), where they are believed to become reactive and contribute to neuroinflammation. A key feature of this reactive gliosis is the secretion of inflammatory mediators. Although in some instances this can be protective, in others, secretion of inflammatory mediators can be harmful, thus possibly contributing to AD pathology. At present, there remains a limited understanding of global astrocytic membrane and extracellular protein profiles and potential AD‐associated changes.

**Method:**

Here, we aimed to address this using a proximity labelling‐based approach. Specifically, we used a viral construct containing TurboID targetted to the ER under a GFAP promoter in order to study astrocyte‐specific proteins trafficked through the classical secretory pathway in an AD mouse model.

**Result:**

After characterising the construct, we investigated changes in proteins between AD and controls over time in response to amyloid pathology. We have identified protein changes which are now being validated using mammalian biofluids such as CSF and plasma.

**Conclusion:**

This work enables a better understanding of astrocyte‐specific membrane and extracellular protein changes as the disease progresses in a mammalian model of AD. An enhanced understanding of this will not only provide insight into astrocyte biology more generally, but may ultimately be vital for identification of novel biomarkers and therapeutic targets for detecting and treating AD.

